# p38 activation occurs mainly in microglia in the P301S Tauopathy mouse model

**DOI:** 10.1038/s41598-022-05980-8

**Published:** 2022-02-08

**Authors:** Juan R. Perea, Esther García, Laura Vallés-Saiz, Raquel Cuadros, Félix Hernández, Marta Bolós, Jesús Avila

**Affiliations:** 1 Centro de Biología Molecular “Severo Ochoa”, Universidad Autónoma de Madrid (UAM-CSIC) (Campus de Cantoblanco), 1 Nicolás Cabrera st, 28049 Madrid, Spain; 2 Center for Networked Biomedical Research On Neurodegenerative Diseases (CIBERNED), 28031 Madrid, Spain; 3 Department of Molecular Biology, Faculty of Sciences, Universidad Autónoma de Madrid, 28049 Madrid, Spain

**Keywords:** Microglia, Cellular neuroscience, Alzheimer's disease, Molecular neuroscience, Neurodegeneration, Inflammation

## Abstract

Tauopathies are a group of neurodegenerative diseases characterized by the accumulation of hyperphosphorylated tau protein in the brain. Many of these pathologies also present an inflammatory component determined by the activation of microglia, the resident immune cells of the brain. p38 MAPK is one of the molecular pathways involved in neuroinflammation. Although this kinase is expressed mainly in glia, its activation in certain neurodegenerative diseases such as Alzheimer's Disease has been associated with its ability to phosphorylate tau in neurons. Using the P301S Tauopathy mouse model, here we show that p38 activation increases during aging and that this occurs mainly in microglia of the hippocampus rather than in neurons. Furthermore, we have observed that these mice present an activated microglial variant called rod microglia. Interestingly, p38 activation in this subpopulation of microglia is decreased. On the basis of our findings, we propose that rod microglia might have a neuroprotective phenotype in the context of tau pathology.

## Introduction

Tau is a microtubule-associated protein that regulates microtubule assembly and stabilization^1^. It is expressed mainly in neurons of the central nervous system (CNS)^2,3^ and is subjected to distinct post-translational modifications^4^. Of these, phosphorylation is the most recurrent and it is regulated throughout lifespan, being more phosphorylated during embryonic development than in adulthood^5,6^. However, in some neurodegenerative pathologies (known as tauopathies), tau is hyperphosphorylated. This fact plays a key role in the physiological function of tau, since it reduces its binding affinity to microtubules and promotes its own aggregation, thus compromising the integrity of neurons^7,8^. Moreover, tau can be released to the extracellular space via various mechanisms, where it interacts with other neurons^9–12^ or glial cells such as astrocytes^13,14^ and microglia^15,16^.

Microglia were first described by the Spanish scientist Pío del Río Hortega in 1919^17^. These cells are the resident macrophages of the CNS^18,19^ and they show basal motility characterized by the continuous extension and retraction of their processes, which allow them to rapidly detect alterations in their microenvironment^20,21^. In Alzheimer’s Disease (AD), the most prevalent tauopathy, a decrease in the neuroprotective functions of microglia, an increase in their toxicity, and alterations in their response to certain stimuli promote the progression of the pathology^22^. These age-associated changes have been previously characterized and include alterations in cytokine secretion^23^, increased expression of activation markers^24^ and the appearance of several microglial morphologies^25–27^. Of the latter, rod microglia are one of the least studied phenotypes to date, despite being present in a wide range of pathologies. Rod microglia are considered an activated microglial variant and they are characterized by elongated cell bodies with processes that project primarily from the apical and basal ends^28,29^. Transcriptomic analysis, along with other techniques, has revealed that microglial activation is a highly complex process and that a wide variety of subpopulations are involved in pathologies with a neuroinflammatory component^30–32^. The aforementioned studies, together with histopathological analysis and neuroimaging results in humans, indicate that the inflammatory response is a phenomenon that occurs simultaneously to Aβ and tau pathology^33,34^. Therefore, it is proposed that neuroinflammation could be the third key factor in the development of AD^35^.

In this regard, p38 MAPK is one of the most important signaling pathways in inflammation^36^. This subfamily comprises four isoforms encoded by different genes, namely p38α (*MAPK14*), p38β (*MAPK11*), p38γ (*MAPK12*) and p38δ (*MAPK13*)^37^. Hensley et al. first described p38 activation in AD. Specifically, they observed an increase in p38 activity in neurons associated with the presence of neurofibrillary tangles and paired helical filaments in the hippocampus^38^. This observation was attributed to the ability of some isoforms (especially p38δ) to phosphorylate tau^39–42^. In this context, most studies to date in this field have focused on the role of p38 in neurons^43^, while its function in microglia has not been revealed.

To address this issue, here we used the P301S Tauopathy mouse model, which overexpresses the 1N4R isoform of human tau with P301S mutation and also presents hallmarks of neuroinflammation^44^. Other authors have made substantial contributions to elucidating the role of microglia in the progression of tau pathology employing this animal model^45,46^. However, studies analyzing p38 activation (a critical trigger of neuroinflammation) are lacking in this mouse. Here we observed that p38 activity in the hippocampus increases with age and that this activation is restricted mainly to microglia, thus highlighting the relevance of p38 in the inflammatory response driven by these cells. Furthermore, we report the presence of rod microglia in this animal model. Surprisingly, rod microglia showed lower levels of p38 activation, suggesting that this microglial variant could have a less activated phenotype and possibly a neuroprotective function.

## Results

### p38 activation increases with age in the P301S mouse hippocampus

**Figure 1:**
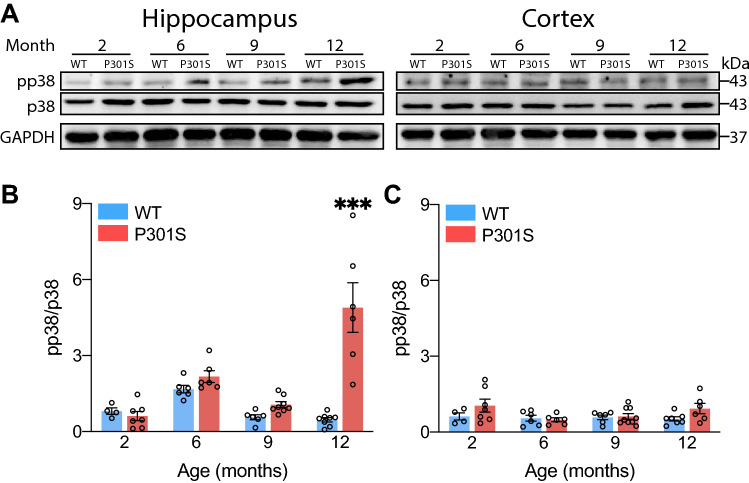
p38 is phosphorylated in the hippocampus of the P301S mouse. Western blot (**A**) and quantification of p38 activity in the hippocampus (**B**) and cortex (**C**) of 2-, 6-, 9- and 12-month-old WT and P301S mice. *n* = 51 (4–8 mice per group). Graphs show mean ± SEM. ***p < 0.001 from two-way ANOVA.

We previously reported that tau protein induces p38 activation in primary cell cultures of microglia and also in the hippocampus of wild-type (WT) animals subjected to stereotaxic injection of tau^47^. However, p38 activation in tauopathy models remains poorly understood. To address this knowledge gap, here we made use of the P301S mouse. This animal shows a progressive accumulation of tau throughout various regions of the brain (entorhinal cortex, hippocampus, neocortex, amygdala, brainstem and spinal cord) in the absence of senile plaques. At late stages, they show neuronal degeneration, ventricular dilatation and signs of neuroinflammation, especially in the cortex and hippocampus^44^. Accordingly, we analyzed p38 activity in these two areas of the brain by western blot, showing that this kinase was more phosphorylated in the latter structure (Fig. 1A). In addition, the increase in phosphorylation was much more pronounced in the hippocampus of 12-month-old P301S animals compared with the other groups and age-matched control (Fig. 1B). At the same time, this increase was positively correlated with phosphorylated tau levels (AT8 and PHF-1) (Supplementary Fig. S1 online). In contrast, we did not find significant differences in the cortex (Fig. 1C).

### Dynamics of p38 activation at the cellular level in the hippocampus and cortex of the P301S mouse

p38 activation in patients with AD has been attributed mainly to neurons^38^, thus being related to tau phosphorylation^39–42^. However, transcriptomic analysis of the diverse cell populations in the CNS reveals that the genes encoding for the different p38 isoforms show high expression in glia^2,3^. Therefore, we used immunofluorescence to study p38 activity in microglia, astrocytes and neurons in the hippocampus and cortex of P301S mice.

**Figure 2:**
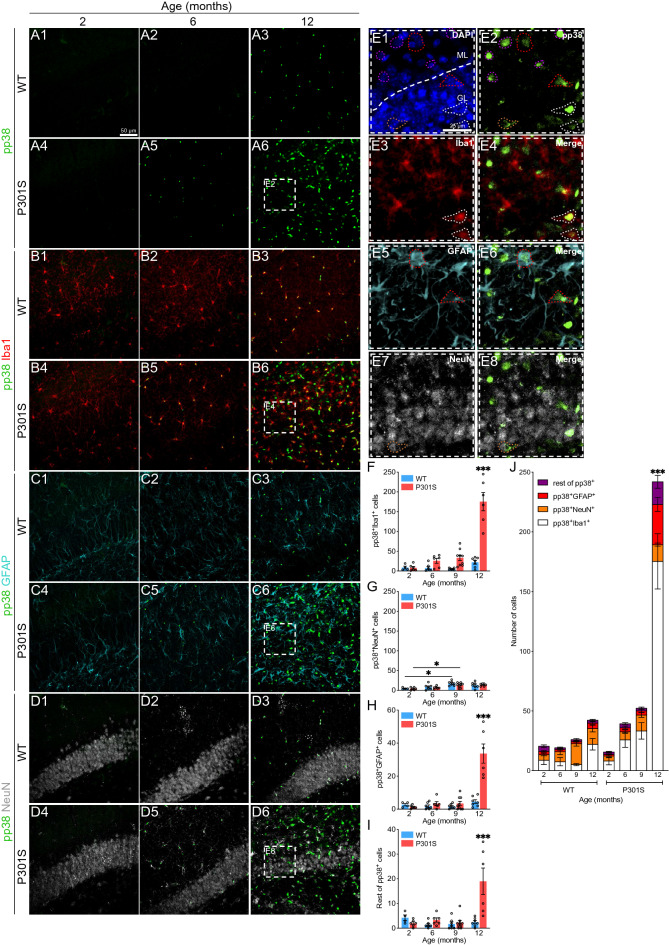
Analysis of p38 activation in the hippocampus of the P301S mouse. (**A-D**) Representative images showing p38 activation (pp38. Green), microglia (Iba1. Red), astrocytes (GFAP. Cyan) and neurons (NeuN. White) in the hippocampus of WT and P301S animals from 2 to 12 months of age. White dashed boxes correspond to the amplified section (**E**). Dashed forms indicate pp38 colocalization with the different cell markers: pp38^+^Iba1^+^ (white), pp38^+^GFAP^+^ (red), pp38^+^NeuN^+^ (orange) and pp38^+^Iba^−^GFAP^−^NeuN^−^ (purple). Nuclei were labeled with DAPI (blue). Number of pp38^+^ cells in microglia (**F**), neurons (**G**), astrocytes (**H**) and remaining cells (**I**). (**J**) Representation of all cell populations together. *n* = 57 (6–9 mice per group). Graphs show mean ± SEM. *p < 0.05; ***p < 0.001 from two-way ANOVA. Scale bars: 50 µm (A1) and 25 µm (E1). GL: granular layer, ML: molecular layer.

Regarding phospho-p38 (pp38) in the hippocampus (Fig. 2A-J), we detected an increase in the activity of this protein, as previously observed by western blot (Fig. 1A-B). By simultaneously observing pp38 and Iba1 (Fig. 2B), we identified significantly more pp38^+^Iba1^+^ cells in the 12-month-old P301S cohort of mice compared with the rest of the groups (Fig. 2F). Similarly, when visualizing pp38 together with GFAP (Fig. 2C), the number of pp38^+^GFAP^+^ cells was also extensive (Fig. 2H). This result could be related to the fact that extracellular tau was able to induce p38 activation in primary cultures of astrocytes (Supplementary Fig. S2 online). Surprisingly, we did not find the same increment in pp38^+^NeuN^+^ cells (Fig. 2D and G), despite the fact that, among other functions, p38 is one of the kinases that regulate tau phosphorylation in neurons^43^. Moreover, we found a population of pp38^+^Iba1^−^GFAP^−^NeuN^−^ cells that are increased in 12-month-old P301S mice (Fig. 2I). The representation of all cell types together revealed that p38 activation, both in microglia and astrocytes, was much more frequent than in neurons or other cell populations in the hippocampus of 12-month-old P301S mice (Fig. 2J).

**Figure 3:**
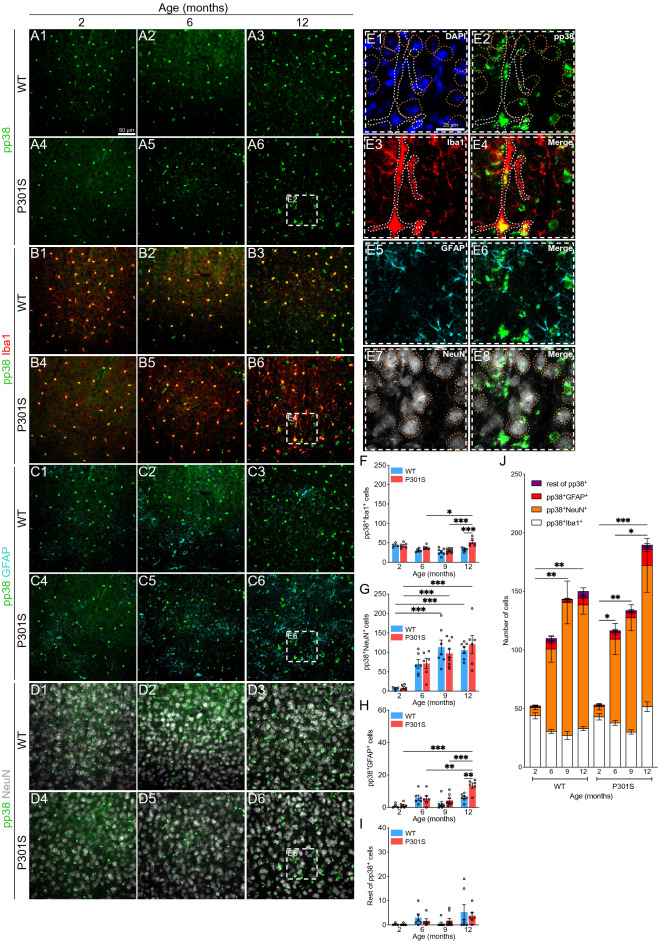
Analysis of p38 activation in the cortex of the P301S mouse. (**A-D**) Representative images showing p38 activation (pp38. Green), microglia (Iba1. Red), astrocytes (GFAP. Cyan) and neurons (NeuN. White) in the cortex of WT and P301S animals from 2 to 12 months of age. White dashed boxes correspond to the amplified section (**E**). Dashed forms indicate pp38 colocalization with the different cell markers: pp38^+^Iba1^+^ (white) and pp38^+^NeuN^+^ (orange). Nuclei were labeled with DAPI (blue). Number of pp38^+^ cells in microglia (**F**), neurons (**G**), astrocytes (**H**) and remaining cells (**I**). (**J**) Representation of all cell populations together. *n* = 57 (6–9 mice per group). Graphs show mean ± SEM. *p < 0.05; **p < 0.01; ***p < 0.001 from two-way ANOVA. Scale bars: 50 µm (A1) and 25 µm (E1).

We next performed the same analysis in the cortex (Fig. 3A-J), identifying a slight increase in pp38^+^Iba1^+^ (Fig. 3B and F) and pp38^+^GFAP^+^ cells (Fig. 3C and H) compared with the hippocampal region. However, the number of pp38^+^NeuN^+^ cells (Fig. 3D and G) showed a significant increase in this area. Consequently, we observed an increase in the number of pp38^+^ cells in the cortex (most of them neurons) throughout the lifetime of these mice, with no statistically significant differences between genotypes (Fig. 3J).

### p38 activation occurs mainly in microglia of the hippocampus

**Figure 4:**
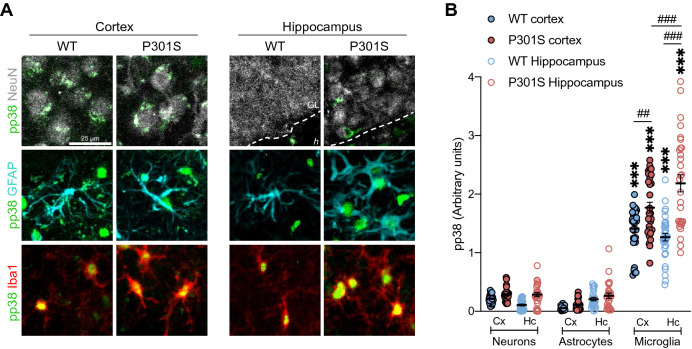
p38 activation occurs mainly in microglial cells of the hippocampus of the P301S mouse. (**A**) Representative images showing p38 activation (pp38. Green) in different cell populations (neurons (NeuN. White), astrocytes (GFAP. Cyan) and microglia (Iba1. Red)) of the cortex and hippocampus of 12-month-old animals. Note that pp38 shows punctate staining in the soma of neurons while it presents a strong signal in the nucleus of glia, especially microglia. (**B**) Quantification of pp38 intensity in each cell population. *n* = 13 (6–7 mice per group). Graph shows mean ± SEM. ^##^*p* < 0.01; ^###^*p* < 0.001; ***p < 0.001 from two-way ANOVA. ^†††^*p* < 0.001 from Student’s *t*-test (two-tailed). Scale bar: 25 µm. GL: granular layer, *h*: *hilus*.

Comparison of the number of pp38^+^ cells in the hippocampus (Fig. 2J) and cortex (Fig. 3J) revealed an increase in these cells in the latter structure. However, western blot analysis showed that p38 activation occurred predominantly in the hippocampus (Fig. 1A-B). To solve this discrepancy, we quantified pp38 intensity as a measure of p38 activity in each cell population individually in 12-month-old mice, showing that p38 was found to be strongly activated in microglia rather than in neurons or astrocytes (Fig. 4A). These differences were more marked in microglia of the P301S hippocampus compared with the cortex (Fig. 4B). This evidence, together with the significant increase of pp38^+^Iba1^+^ cells in the hippocampus of P301S mice (Fig. 2F and J), could explain the strong p38 activation in this structure identified by western blot (Fig. 1A-B).

Besides, the analysis of pp38 intensity in NeuN^+^ cells showed that p38 activation was increased (*t* = 2.881; *p* < 0.0001) in the neurons of the hippocampus of P301S mice compared to WT (Fig. 4). These data further confirmed the results obtained by western blot (Fig. 1A-B) and were related to the increase in tau phosphorylation (AT8 and PHF-1) in the hippocampal region (Supplementary Fig. S1 online). Aside from that, a recent research has demonstrated an additional function of p38 in neurons beyond tau phosphorylation, showing that p38 activation has an essential role in the proliferation of neural progenitor cells^48^. Concretely, this study demonstrated that p38 signaling represses the expression of *Dkk1* and *Sfrp3* genes that are involved in adult hippocampal neurogenesis. In this sense, qPCR analysis also showed that *Dkk1* was downregulated in the hippocampus of P301S mice compared to WT (Supplementary Fig. S3 online).

### Rod microglia in the P301S mouse show lower levels of p38 activation

**Figure 5:**
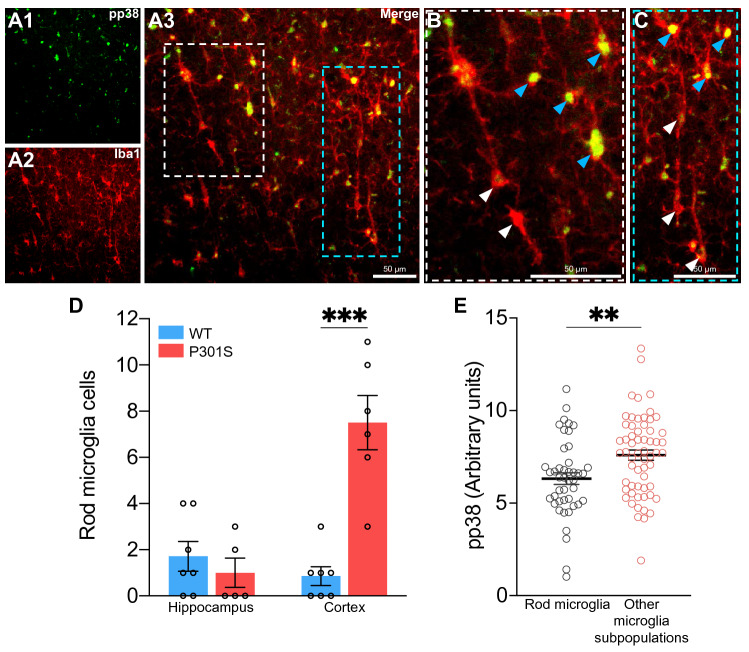
Rod microglia in the P301S mouse show lower levels of p38 activation. (**A**) Representative image of rod microglia in the cortex of a 12-month-old P301S mouse. White and blue dashed boxes correspond to the amplified sections (**B** and **C**). Note that rod microglia show lower levels of pp38 (white arrows) compared with other microglial subpopulations (blue arrows). (**D**) Number of rod microglia in the hippocampus and cortex of 12-month-old mice. ***p < 0.001 from two-way ANOVA. (**E**) Quantification of pp38 intensity in rod microglia. **p < 0.01 from Student’s *t*-test (two-tailed). *n* = 13 (6–7 mice per group). Graphs show mean ± SEM. Scale bars: 50 µm.

A series of microglial subpopulations have been described on the basis of their morphology ^27^. Of these, it has been shown that rod microglia appear more frequently during aging^49^, after traumatic brain injury^50^ and in several neurodegenerative diseases^27,51–53^. In this regard, previous analysis carried out in this study allowed us to identify the presence of rod microglia in 12-month-old P301S mice (Fig. 5A), and more specifically in the cortex (Fig. 5D). Given that the role of rod microglia is still unclear and it is not known whether they exert a toxic or neuroprotective function^29^, we sought to analyze p38 activation in these cells. We found that p38 activity in rod microglia was decreased compared with the other microglial subpopulations (Fig. 5B, C and E). Accordingly, and taking into account that p38 activation is related to inflammatory processes^36^, rod microglia might have a less activated phenotype.

## Discussion

After the discovery of p38, the first studies in this field associated the activation of this kinase with the inflammatory response against harmful stimuli^54,55^. However, in recent years, research into its role in the CNS has been focused on tau phosphorylation in neurons^56–58^, overlooking additional functions in other cell types. Nonetheless, the increased activation of p38 in the brains of various mouse models could be due to its activation in glia, which show high levels of its transcript^2,3^. To solve this question, we analyzed p38 activity in the three main cell types of the brain (neurons, astrocytes and microglia) in WT and P301S mice throughout aging. We reported that p38 activation increased with age in the hippocampus (especially in P301S mice), and this activation occurred mainly in microglia. In addition to the increased microgliosis observed in P301S mice, we identified the presence of an activated microglial variant (rod microglia) in the cortex of this model that is normally present in the aged and diseased brain^29^. Surprisingly, rod microglia showed low levels of p38 activation, which could partly explain the possible neuroprotective role of this subpopulation. All these results suggest that p38 in microglial cells plays a key role in the progression of neurodegenerative diseases such as tauopathies, particularly in the inflammatory component of these conditions.

Previous research have reported α-synuclein and Aβ toxic proteins activate p38 in microglia^59,60^, while we have shown tau induces p38 activation and subsequent inflammatory response in primary microglia cultures^47^. Furthermore, that study also showed that tau inoculation in the hippocampus of WT mice increased p38 activation in this region^47^. In the present study, using the P301S Tauopathy mouse model, we also observed an increase in p38 activation in the hippocampus. This phenomenon has been described by others^38,61^. However, despite proposing that part of the p38 phosphorylation takes place in glia, this notion was never supported using specific markers. In line with this, our results describe for the first time that microglia of P301S animals show high levels of p38 activation compared with other cell populations of the brain. Additionally, a more exhaustive analysis allowed us to distinguish that p38 activation is higher in microglia of the hippocampus than in the cortex of the P301S mouse. This observation might be related to the early appearance of hyperphosphorylated tau in this region and the consequent neuroinflammation^44,62^, which gives rise to a series of distinct microglial morphologies. In this regard, we identified the presence of an activated microglial variant—rod microglia—observed in several pathological conditions.

Rod microglia present an elongated soma with thin processes and they are aligned with the dendrites of neighboring neurons^28^. While other microglial morphologies are associated with a specific function, such as ramified for surveying the brain parenchyma, the role of rod microglia remains poorly understood. In the context of tau pathology, our results demonstrate the presence of rod microglia in the cortex of the P301S mouse. Other authors also reported that this microglia variant was present in tauopathy murine models such as Tx5 mouse (5xfAD crossed with Thy-Tau22)^63^ and R926-hTau rat^64^. In addition to this, it has been shown that tau inoculation in the brain of mice caused the appearance of rod microglia, which was in the vicinity of AT8^+^ neurons^65–67^. This phenomenon has also been observed close to PHF-1^+^ neurons in AD^27^ and Down syndrome patients^68^. Those findings are in agreement with recent observations by Woollacott et al., who determined the presence of rod microglia in the cortex of patients with frontotemporal lobar degeneration with tau pathology (FTLD-Tau)^52^. Our data, along with the aforementioned studies, provide solid evidence regarding the presence of rod microglia in Tauopathies. Surprisingly, only 40% of the P301S mice presented rod microglia in the hippocampus (the most affected region in this model), with an average number of 2 to 3 cells/animal. In line with this, Bachstetter et al. reported similar results in patients with AD, dementia with Lewy bodies and hippocampal sclerosis with aging, where no more than 60% of the patients presented rod microglia in the hippocampus, with an average number of cells/case very close to our results (2.1 to 3.9)^27^. Consequently, this body of evidence could explain the lower presence of rod microglia in the hippocampus.

Furthermore, we observed that p38 activity is decreased in rod microglia in the P301S mouse. Given that: (i) p38 activation has been linked to innate immunity and inflammation processes^69,70^; (ii) extracellular tau activates p38 in microglia, triggering a pro-inflammatory response ^47^; and (iii) rod microglia have lower levels of pro-inflammatory cytokines than ameboid microglia^71^, we propose that rod microglia could exert a neuroprotective function. In this sense, tau immunotherapy in progressive supranuclear palsy (PSP) patients with Gosuranemab was associated with a glial response characterized by numerous rod microglia processes^72^. Thus, we hypothesize this treatment strategy could shift microglia ﻿shift towards a less activated state. However, as most of the studies focused on rod microglia have been limited to histological samples, future endeavors should provide an in-depth analysis of the molecular features of this subpopulation. For example, fate-mapping techniques^73^, together with in vivo imaging technologies and omics approaches, such as spatial transcriptomics^74^, would help decipher the role of rod microglia in health and disease with greater precision^29^.

Regarding other glia, the increased number of pp38^+^ astrocytes in the hippocampus and cortex of the P301S mouse correlates with the astrogliosis observed in this model^44^. Accordingly, Lo et al. demonstrated that p38α is involved in the inflammation response driven by astrocytes^75^. In fact, our previous results revealed that extracellular tau induces p38 activation in cultured astrocytes. In addition to the function of p38 in the innate immune system, other authors have recently reported that astrocytic p38α is essential for long-term depression (LTD) and that it modulates long-term memory^76^. Although the P301S mouse exhibits impaired long-term potentiation (LTP)^44,77^, the consequences of astrocytic p38 activation on this form of synaptic plasticity remains unexplored in this model.

Moreover, we found a population of pp38^+^Iba1^−^GFAP^−^NeuN^−^ cells that are increased in the hippocampus of P301S mice. Previous studies performed with human samples and other transgenic models have revealed that p38 signaling takes place in other cell types of the brain. In this regard, p38 is highly expressed in pericytes and endothelial cells, contributing to neuroinflammation and playing an important role in the integrity of the blood–brain barrier^78,79^. Moreover, p38 activity has also been reported in oligodendrocytes of patients with distinct tauopathies^80,81^. Further research should be focused on deciphering the identity of these cells in the P301S mouse model, as well as elucidating whether p38 activity occurs in the microglia of patients with tau pathology.

Additionally, the number of pp38^+^ neurons increased progressively during aging both in P301S mice and their littermates. A more specific analysis carried out herein has revealed that p38 activity is higher in hippocampal neurons of P301S mice compared with WT counterparts. These results are in agreement with recent studies performed by Moreno-Cugnon et al., reporting that p38 activity increases in neurons with age, thus being detrimental to neuronal function^82^. Furthermore, this enhanced activity of p38 in hippocampal neurons, as well as its expression pattern (dotted points in the soma of neurons *vs.* nuclear staining in glia), supports the involvement of this kinase in tau phosphorylation, as numerous authors have previously reported^39–42,83^. In addition to its role in tau phosphorylation, p38 signaling is essential for the proliferation of neural progenitor cells and, therefore, for the maintenance of adult neurogenesis during aging^48^. The authors of that study found that p38 suppresses the expression of *Dkk1* and *Sfrp3*, molecules that are antagonists of the Wnt signaling pathway, which promotes adult hippocampal neurogenesis^48,84,85^. In this context, we report that P301S mice present lower levels of *Dkk1* and hypothesize that this model attempts to compensate for the marked neuronal loss that occurs in the hippocampal region. Given that adult hippocampal neurogenesis has not been studied in this model of tauopathy^86^, future lines of research should be focused on this subject.

In summary, our results reveal that p38 activation is greater in microglia compared with other cell populations in the aging brain of the P301S mouse. Hence, the p38 MAPK pathway may have greater relevance in the inflammatory processes that occur in tauopathies than its role in neurons, such as tau phosphorylation. On the basis of our findings, and given that p38 inhibition exerted positive effects in several mouse models of AD^87–89^, the targeted deletion/inhibition of p38 in microglia emerges as a promising therapeutic strategy to halt neuroinflammation during tau pathology.

## Methods

### Animals and genotyping

The P301S Tauopathy mouse model (B6;C3-Tg(Prnp-MAPT*P301S)PS19Vle/J) was generated by Yoshiyama et al. and donated to The Jackson Laboratory (stock number 008169). The line was kept in hemizygosis by back-crossing hemizygous males with C57BL/6J females since homozygous females do not mate. In this way, the B6C3F1 genetic background of the first generation was homogenized to C57BL/6J for fifteen generations. These animals express the 1N4R isoform of human tau with the P301S mutation under the expression of the prion protein (*Prnp*) promoter. The expression of mutated human tau in these animals is five times higher than endogenous tau^44^.

All mice were housed at the animal facility of the “Centro de Biología Molecular Severo Ochoa”. They had access to food and water ad libitum and were maintained in a temperature-controlled environment on a 12/12 h light/dark cycle. Animal housing and maintenance protocols followed the guidelines of the Council of Europe Convention ETS123, revised as indicated in the Directive 86/609/EEC. Animal experiments were performed under protocols (P15/P16/P18/P22) approved by the Institutional Animal Care and Utilization Committee (Comité de Ética de Experimentación Animal del CBM, CEEA-CBM, Madrid, Spain) and following ARRIVE guidelines.

The genotype of this model was determined by nested PCR from genomic DNA obtained from a small portion of the tail. To this end, the tissue was digested with 150 µl of 50 mM NaOH in a thermomixer (1 h, 450 rpm, 99 °C). The digestion was neutralized with 15 µl of 1 M Tris–HCl pH 8 and subsequently centrifuged (10 min, 18,400 g, room temperature). The components of the GoTaq G2 Flexi DNA Polymerase kit (Promega), the dNTP mix (Thermo Fisher) and the primers (Supplementary Table S1 online) were added as follows: 2 µl of genomic DNA; 2.4 μl of 5 × green buffer; 0.96 µl of 25 mM MgCl_2_; 0.48 µl of 5 mM dNTP mix; 0.3 µl of each of the 20 µM primers; 0.1 µl Taq polymerase (5U/µl); and Milli-Q water up to 10 μl volume. Nested PCR has 2 rounds of amplification after an initial time of 3 min at 94 °C. The first round of amplification consists of 11 cycles of denaturation (94 °C, 15 s), annealing (72 °C, 15 s) and extension (72 °C, 10 s). The second round consists of 29 cycles of denaturation (94 °C, 15 s), annealing (65 °C, 15 s) and extension (72 °C, 10 s). Finally, the elongation time is for 10 s at 72 °C. The PCR product results in a 450 bp band (transgene region) and a 200 bp band (murine gene region).

### Tissue sample preparation

Animals were anesthetized by intraperitoneal injection of sodium pentobarbital (60 mg/kg) (Dolethal. Vetoquinol) and subsequently perfused intracardially with 50 ml of 0.9% saline solution. Next, the brain was removed and the two hemispheres were separated.

The hemispheres used for immunofluorescence were fixed in 4% paraformaldehyde (Electron Microscopy Sciences) overnight at 4 °C with mild shaking. The following day, they were washed 3 times with 0.1 N PB and later included in 10% sucrose and 4% agarose in 0.1 N PB. Next, blocks were cut into coronal sections of 50 μm thickness in a VT1200S vibratome (Leica). Samples were stored at −20 °C in a cryoprotective solution composed of 30% ethylene glycol and 26% glycerol in 0.1 N PB.

The hippocampus and cortex from the other hemisphere were dissected, immediately frozen in dry ice, and stored at −80 °C for biochemical analysis. At the time of use, samples were homogenized on ice using RIPA buffer (50 mM Tris–HCl pH 7.4; 150 mM NaCl; 1% Triton X-100; 0.5% sodium deoxycholate; 0,1% SDS) with a mixture of protease (cOmplete. Roche) and phosphatases (0.1 mM okadaic acid and 5 mM orthovanadate) inhibitors. After a centrifugation step (5 min, 845 g, 4 °C), the protein concentration of the supernatant was determined by BCA assay (Thermo Fisher).

### Western blot

5 × Laemmli buffer (0.125 M Tris–HCl pH 6.8; 20% glycerol; 5% SDS; 0.004% bromophenol blue; 2% β-mercaptoethanol) was added to the protein extracts, which were denatured at 100 °C for 5 min. 30 μg of proteins were then separated by standard SDS-PAGE electrophoresis in 10% polyacrylamide gels. They were then transferred to a 0.45 μm nitrocellulose membrane (GE Healthcare) using the Mini-PROTEAN system (Bio-Rad) at a constant amperage of 150 mA for 50 min. The membrane was blocked with 5% BSA in 0.1% TBS-T for 2 h at room temperature and incubated overnight at 4 °C with primary antibodies. The following day, the membrane was washed 3 times with 0.1% TBS-T and incubated with secondary antibodies for 2 h at room temperature. Finally, the membrane was subjected to 3 washes with 0.1% TBS-T before being developed with ECL (PerkinElmer) and photographed on an ImageQuant LAS 4000 mini system (GE Healthcare). The signal intensity was quantified by densitometry using Fiji. To correct for possible loading errors, the densitometry values ​​obtained were normalized with loading controls.

### Immunofluorescence

Tissue sections were washed 3 times with 0.1 N PB to remove the cryoprotectant solution. They were then incubated with primary antibodies diluted in the blocking solution (1% BSA and 1% Triton X-100 in 0.1 N PB) for 48–72 h at 4 °C. After 5 washes in blocking solution, samples were incubated overnight at 4 °C in darkness with the fluorescent secondary antibodies. The next day, after 3 washes with 0.1 N PB, the nuclei were labeled with 1 µg/ml DAPI (Merck) for 10 min at room temperature in darkness. After another 3 washes with 0.1 N PB, sections were mounted on gelatinized slides.

Images were obtained using the A1R + confocal system coupled to an Eclipse Ti-E inverted microscope (Nikon) with a 40 × objective. We used a spectral detector for the simultaneous acquisition of 5 different fluorophores. Next, the signal corresponding to each fluorophore was identified and separated using the unmixing tool of the NIS Elements v4.4 programme (Nikon). To this end, it was necessary to introduce individual controls for each fluorophore.

Each image is an overlay of 25 stacks with a separation of 1.4 μm between them. After subtracting the background signal from each channel, the number of positive cells for each marker was quantified manually using the Cell counter tool of Fiji in a specific field that spanned 625 × 625 μm in *xy* and 35 μm in depth. The mean value of the selected zone was used to quantify the signal intensity of pp38 in each cell population.

### Statistical analysis

The statistical analysis of the data was carried out using GraphPad Prism v.9.0.0. The presence of outliers was checked using the Grubbs test. Subsequently, Shapiro–Wilk and D’Agostino-Pearson tests were used to verify that the remaining values ​​were adjusted to a normal distribution. For the comparison between two experimental groups, data were analyzed by Student’s *t*-test (two-tailed). Two-way analysis of variance (ANOVA) tests were applied to compare more than two experimental groups, and post hoc comparisons were performed using Tukey’s multiple comparison test. To estimate the relationship between variables, a linear regression analysis was carried out in which we calculated the coefficient of determination (R^2^). Details of sample size (*n*), data representation, the statistical test used, and significance levels are provided in the caption of each figure.

## Supplementary Information

Supplementary Information.

## Data Availability

The data that support the findings of this study are available from the corresponding author upon request.
