# Supplementary material for: p38 activation occurs mainly in microglia in the P301S Tauopathy mouse model
Source: Sci Rep. 2022 Feb 8;12:2130. doi: 10.1038/s41598-022-05980-8 (PMC8826411; doi:10.1038/s41598-022-05980-8)
Supplement: Supplementary file 1 — Supplementary Information. [file 41598_2022_5980_MOESM1_ESM.pdf]

Supplementary information for:

p38 activation occurs mainly in microglia in the P301S Tauopathy mouse model

Perea JR[1,2], García E[1], Vallés-Saiz L[1], Cuadros R[1], Hernández F[1,2,3], Bolós M[1,2], Avila J[1,2,*]

[1]Department of Molecular Neuropathology, Centro de Biología Molecular "Severo Ochoa" (UAM-CSIC), Madrid 28049, Spain
[2]Center for Networked Biomedical Research on Neurodegenerative Diseases (CIBERNED), Madrid 28031, Spain
[3]Department of Molecular Biology, Faculty of Sciences, Universidad Autónoma de Madrid, Madrid 28049, Spain
[*]Correspondence should be addressed to Jesús Avila. Centro de Biología Molecular "Severo Ochoa", Universidad Autónoma de Madrid (Campus de Cantoblanco), 1 Nicolás Cabrera st, 28049, Madrid (Spain). Phone number: +34-911964564. javila@cbm.csic.es

Supplementary figures

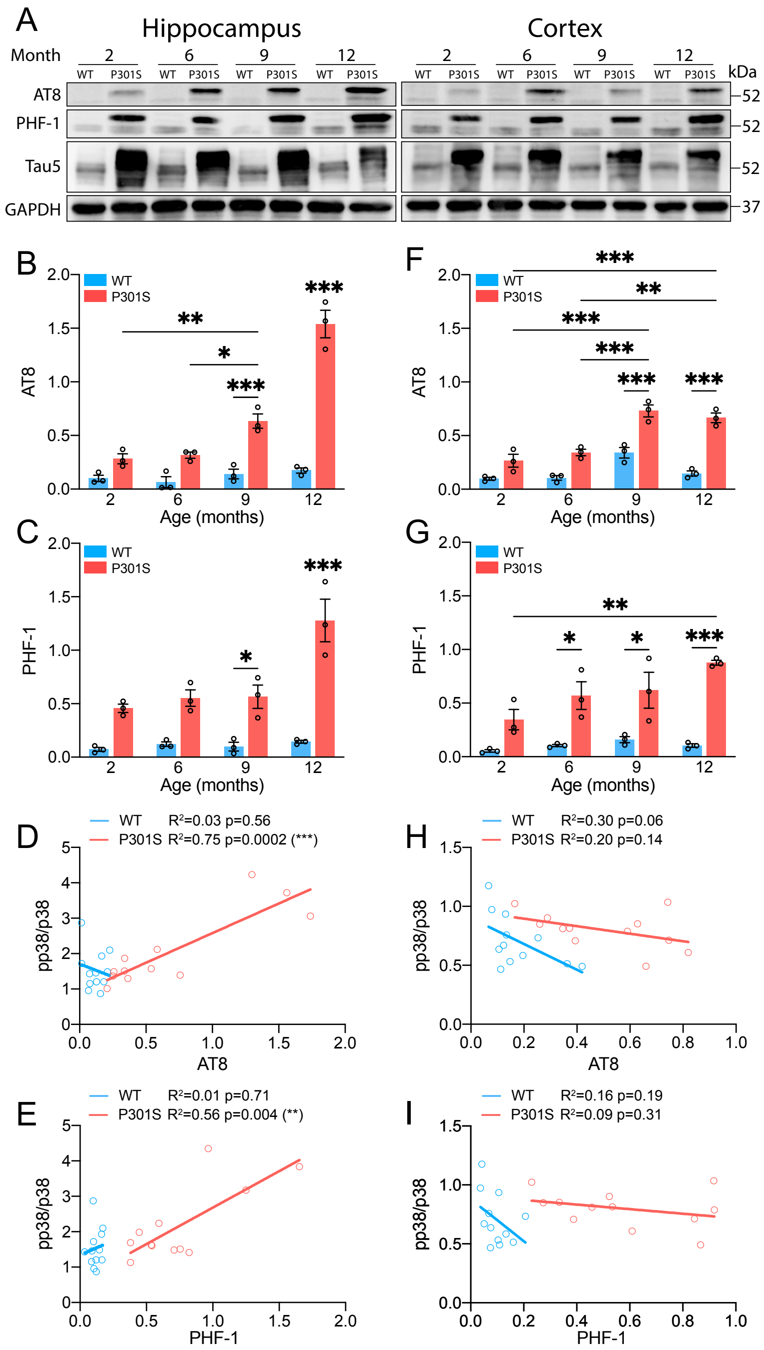

Supplementary Figure S1: Positive correlation between pp38 and phosphorylated tau in the hippocampus of P301S mouse. (A) Representative western blot of phospho-tau (AT8 and PHF-1) and total-tau (Tau5) levels in the hippocampus and cortex of 2-, 6-, 9- and 12-month-old WT and P301S mice. Quantification of AT8 (B) and PHF-1 (C) levels in the hippocampus. Linear regression analysis between pp38 and AT8 (D) or PHF-1 (E) in the hippocampus. Note the positive correlation present in transgenic mice compared to WT controls. Quantification of AT8 (F) and PHF-1 (G) levels in the cortex. Linear regression analysis between pp38 and AT8 (H) or PHF-1 (I) in the cortex. pp38/p38 values are derived from Figure 1. Graphs show mean ± SEM. **p*<0.05; ***p*<0.01; ****p*<0.001 from two-way ANOVA. Linear regression analysis between pp38 and AT8 (D) or PHF-1 (E) levels. ***p*<0.01; ****p*<0.001. *n*=24 (3 mice per group).

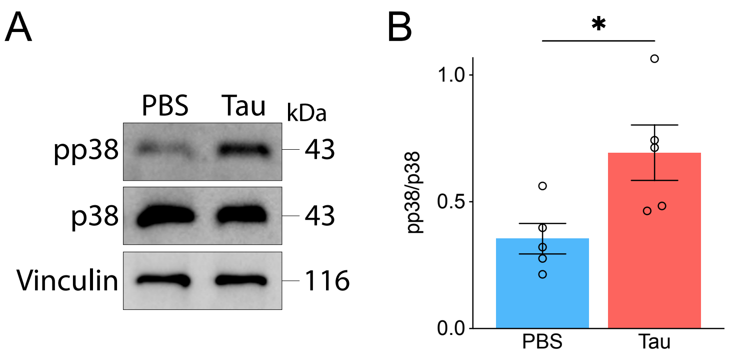

Supplementary Figure S2: Tau activates p38 in astrocytes *in vitro*. Western blot (A) and quantification of pp38 levels (B) in primary astrocytes culture treated with PBS (control) or extracellular monomeric tau for 1 h. Graph shows mean ± SEM. *p<0.05 from Student's t-test (two-tailed). n=5.

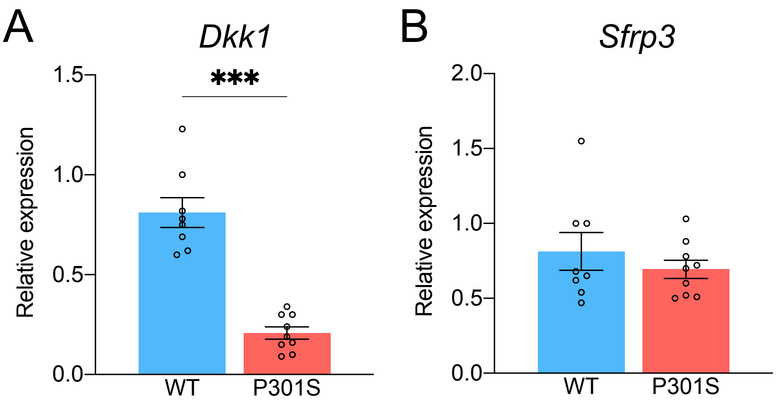

Supplementary Figure S3: *Dkk1* and *Sfrp3* expression levels in the hippocampus 12-month-old P301S mice. Graphs show mean ± SEM. ***p<0.001 from Student's t-test (two-tailed). n=17 (8-9 mice per group).

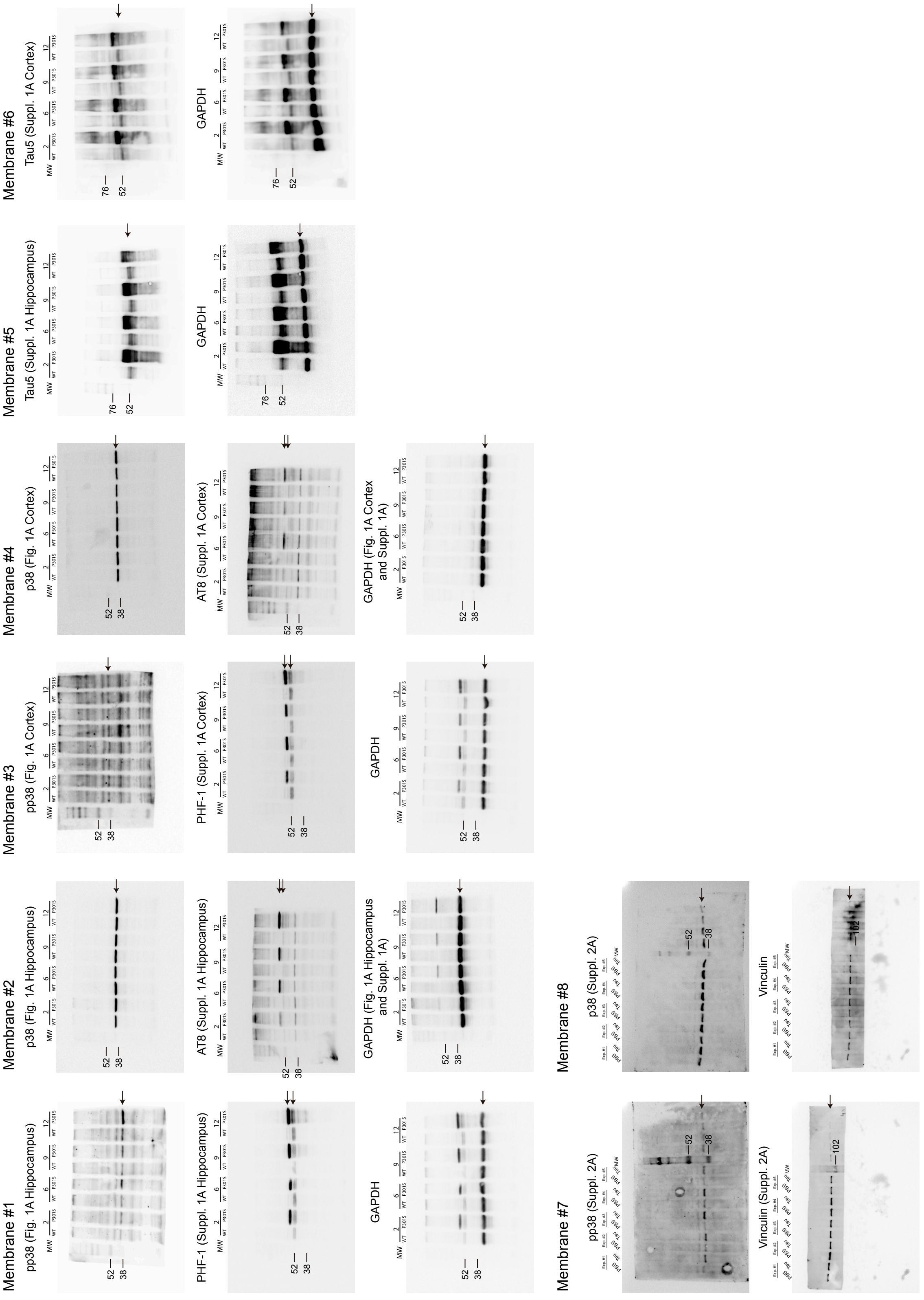

Supplementary Figure S4: Full-length blots of Figure 1A, Supplementary Figure 1A and Supplementary Figure 2A.

Supplementary tables

Genotype	Forward	Reverse
**WT**	CAAATGTTGCTTGTCTGGTG	GTCAGTCGAGTGCACAGTTT
**P301S**	GGCATCTCAGCAATGTCTCC	GGTATTAGCCTATGGGGGACAC

Supplementary Table S1: PCR primers for genotyping.

Antibody	Host	Manufacturer (ref.)	Dilution
**Anti-AT8**	Mouse (m)	Thermo Fisher (MN1020)	1:100 (WB)
**Anti-PHF-1**	Mouse (m)	Courtesy of Peter Davies	1:100 (WB)
**Anti-Tau5**	Mouse (m)	Merck (577801)	1:1,000 (WB)
**Anti-pp38**	Rabbit (p)	Cell Signaling (9211)	1:1,000 (WB)
**Anti-pp38**	Rabbit (m)	Cell Signaling (4511)	1:1,600 (IF)
**Anti-p38**	Rabbit (p)	Cell Signaling (9212)	1:1,000 (WB)
**Anti-Iba1**	Guinea pig (p)	Synaptic Systems (234 004)	1:500 (IF)
**Anti-NeuN**	Mouse (m)	Merck (MAB377)	1:500 (IF)
**Anti-GFAP**	Chicken (p)	Abcam (ab4674)	1:2,000 (IF)
**Anti-GAPDH**	Mouse (m)	Abcam (ab8245)	1:1,000 (WB)
**Anti-Vinculin**	Rabbit (m)	Abcam (ab129002)	1:10,000 (WB)
**Anti-mouse HRP**	Goat (p)	Dako (P0447)	1:2,000 (WB)
**Anti-rabbit HRP**	Goat (p)	Dako (P0448)	1:2,000 (WB)
**Anti-rabbit 488**	Donkey (p)	Thermo Fisher (A-21206)	1:1,000 (IF)
**Anti-guinea pig 555**	Goat (p)	Thermo Fisher (A-21435)	1:1,000 (IF)
**Anti-mouse 594**	Donkey (p)	Thermo Fisher (A-21203)	1:1,000 (IF)
**Anti-chicken 647**	Goat (p)	Thermo Fisher (A-21449)	1:1,000 (IF)

Supplementary Table S2: Primary and secondary antibodies. m: monoclonal, p: polyclonal, WB: western blot, IF: immunofluorescence.

Gene	Forward	Reverse
Dkk1	CCGGGAACTACTGCAAAAAT	CCAAGGTTTTCAATGATGCTT
Sfrp3	CACCGTCAATCTTTATACCACCT	TCAGCTATAGAGCCTTCTACCAAGA
Actb	CTAAGGCCAACCGTGAAAAG	ACCAGAGGCATACAGGGACA
Rna18s1	CTCACCACGGGAAACCTCAC	CGCTCCACCACCTAAGAACG

Supplementary Table S3: qRT-PCR primers.

Supplementary Methods

Recombinant tau preparation

pRKT42, which encodes for full-length tau protein of human origin (2N4R), was transformed into LPS Free ClearColi (BL21(DE3)) cells (Lucigen) following the manufacturer's instructions. Single colonies were grown in LB medium with 100 µg/ml ampicillin, and 0.4 mM IPTG was added when OD_{600} reached 0.6-0.8. Four hours later, bacteria were pelleted (20 min, 1,000 *g*, 4°C) and sonicated in extraction buffer (0.1 M MES; 2 mM EGTA; 0.5 mM $MgCl_2$; 0.5 M NaCl; 5 mM β-mercaptoethanol; 1 mM PMSF). The sonicated product was centrifuged (10 min, 23,700 *g*, 4°C) and the supernatant was boiled for 10 min to prevent possible contamination of endogenous enzymatic activities. The boiled sample was incubated on ice for 5 min and subsequently centrifuged (30 min, 23,700 *g*, 4°C). Tris was then added to the supernatant until pH = 11.

To induce tau precipitation, the sample was stirred for 1 h at 4°C in a magnetic mixer and $(NH_4)_2SO_4$ was added until reaching 50% saturation. Next, the sample was centrifuged (1 h, 23,700 *g*, 4°C) and the pellet was resuspended in PBS. The resuspension was dialyzed in PBS with a Spectra/Por3 membrane (Repligen) overnight at 4°C in a magnetic mixer. The next day, the dialyzed product was incubated for 30 min at 37°C while shaking with 10 µg/ml RNase A (Roche). To stop the enzymatic activity of the RNase and remove it, the sample was incubated for 1 min at 100°C and centrifuged (1 min, 15,900 *g*, 4°C).

To efficiently remove the remaining endotoxins, the supernatant was subjected to Triton X-114 phase separation. First, Triton X-114 was added to a concentration of 1% and the sample was incubated for 30 min at 4°C in a treadmill with a vortex step every 5 min. The sample was then incubated for 10 min at 56°C in a water bath and centrifuged (10 min, 20,000 *g*, 25°C) to obtain the liquid phase. This procedure was repeated three times and the liquid phase of the third cycle was processed through Pierce detergent removal spin column (Thermo Fisher) following the manufacturer's instructions. The eluted volume was passed through a Polymyxin B-agarose (Sigma Aldrich) endotoxin removal column, and the endotoxin levels were quantified by LAL assay (HyCult Biotech).

Primary cultures of astrocytes

Astrocytes were cultured from newborn C57BL/6J mice (P0-P3). Brains were dissected in cold Ca^{2+}/Mg^{2+}-free HBSS and stripped of meninges. The tissue was then digested with 0.25% trypsin for 10 min at 37°C. Trypsinization was stopped by the addition of the medium used for this cell culture: DMEM supplemented with 1% Glutamax (Gibco), 10% FBS (Gibco), 100 U/mL of penicillin and 0.1 mg/mL of streptomycin (Gibco). Next, 0.2 mg/ml DNase (Roche) was added to the digested tissue and centrifuged (10 min, 300 *g*, 4°C). A single-cell suspension of the pellet was obtained by repeated pipetting. Subsequently, cells were passed through a 40 µm nylon filter (Corning) and seeded into 75 cm^2 flasks coated with 0.1 mg/ml poly-L-lysine (Sigma Aldrich) (2 brains/flask), which were maintained at 37°C in humidified 5% CO_2–95% air. Next day, the plating medium was discarded, the flasks were washed three times with PBS, and fresh medium was added. After 7-10 days, microglia were discarded by shake

off and astrocytes were seeded on 12-well plates ($2x10^5$ cells/well). Three days later, astrocytes were treated with 0.5 µM Tau or PBS (control) for 30 min. They were then washed three times with PBS to remove excess tau. Finally, the samples were lysed using the RIPA buffer (50 mM Tris-HCl pH = 7.4; 150 mM NaCl; 1% Triton X-100; 0.5% sodium deoxycholate; 0.1% SDS) with a mixture of protease (cOmplete. Roche) and phosphatases (0.1 mM okadaic acid and 5 mM orthovanadate) inhibitors. After a centrifugation step (5 min, 845 *g*, 4°C), the protein concentration of the supernatant was determined by BCA assay (Thermo Fisher).

qRT-PCR

RNA was isolated with the Maxwell 16 miRNA Tissue kit (Promega), and reverse transcription (RT) reaction was performed using the iScript cDNA Synthesis Kit (Bio-Rad), following the manufacturer's protocols in both cases. RT-qPCR of selected genes was performed by using the ABI PRISM 7900HT SDS system (Applied Biosystems). Each reaction contained: 1 µl of the cDNA template to a final concentration of 35 ng, 5 µl of the SYBR Green PCR mix (Applied Biosystems), 1 µl per primer pair at 5 µM (Supplementary Table S3) and 3 µl of RNase-free water. Gene amplification was performed for 40 cycles of 95ºC for 1 s and 60ºC for 20 s. No amplification from the no-template control (NTC) was observed for any of the genes of interest. All the target genes were normalized for relative quantification by the normalization factor (NF) derived from geometric means ΔΔ-Cq (quantification cycles) of 18S (murine ribosomal) and β-actin (*Actb*) mRNA levels. Three technical replicates per gene were made. Each primer pair showed a single and sharp peak, thereby indicating that the primers amplified only one specific PCR product.
